# Optical genome mapping refines diagnosis of high-grade myeloid neoplasms through detection of *MECOM* rearrangements cryptic to conventional karyotype

**DOI:** 10.1093/ajcp/aqag087

**Published:** 2026-08-04

**Authors:** Nicolas Salcedo-Porras, Fatma AlBulushi, Julia N C Parlow, Eric McGinnis, Tara Spence

**Affiliations:** Department of Pathology and Laboratory Medicine, Faculty of Medicine, University of British Columbia, Vancouver, BC, Canada; Cytogenomics Laboratory, Department of Pathology and Laboratory Medicine, Vancouver General Hospital, Vancouver, BC, Canada; Department of Pathology and Laboratory Medicine, Faculty of Medicine, University of British Columbia, Vancouver, BC, Canada; Cytogenomics Laboratory, Department of Pathology and Laboratory Medicine, Vancouver General Hospital, Vancouver, BC, Canada; Department of Pathology and Laboratory Medicine, Faculty of Medicine, University of British Columbia, Vancouver, BC, Canada; Cytogenomics Laboratory, Department of Pathology and Laboratory Medicine, Vancouver General Hospital, Vancouver, BC, Canada; Department of Pathology and Laboratory Medicine, Faculty of Medicine, University of British Columbia, Vancouver, BC, Canada; Cytogenomics Laboratory, Department of Pathology and Laboratory Medicine, Vancouver General Hospital, Vancouver, BC, Canada; Department of Pathology and Laboratory Medicine, Faculty of Medicine, University of British Columbia, Vancouver, BC, Canada; Cytogenomics Laboratory, Department of Pathology and Laboratory Medicine, Vancouver General Hospital, Vancouver, BC, Canada

**Keywords:** optical genome mapping, *MECOM* rearrangement, acute myeloid leukemia, cancer cytogenetics, cryptic rearrangements

## Abstract

**Objectives:**

*MECOM* rearrangements (*MECOM*-r) are high-risk events in myeloid neoplasms often cryptic or incompletely characterized by conventional karyotype analysis. We used optical genome mapping (OGM) to define the frequency and spectrum of *MECOM*-r in an unselected, consecutively collected cohort of individuals with high-grade myeloid neoplasms (HGMNs) and compared these findings with standard cytogenetic approaches.

**Methods:**

A total of 252 HGMN specimens, including 224 with definitive diagnoses of acute myeloid leukemia, were processed over a 2-year period using OGM, G-banded karyotyping and, in some cases, reflex fluorescence in situ hybridization (FISH) for the *MECOM* locus. Cases were retrospectively reviewed to determine whether the identification of *MECOM*-r by OGM refined diagnosis and/or prognosis.

**Results:**

Our diagnostic workflow identified *MECOM*-r in 12 cases (4.7%), only 6 of which were identified by combined karyotype and FISH analyses, the latter for suspected chromosome 3 abnormalities. The remaining 6 cases were cryptic due to normal karyotypes, highly complex karyotypes lacking apparent chromosome 3 involvement, or culture failure and thus were identified by OGM and reflex FISH. Furthermore, OGM clarified complex architectures, resolved genetic alterations, and identified diverse partner loci, including *GATA2*, *THADA*, *MYC*, *PHACTR1*, *MB21D2*, *XR_001744423.2*, *IL12A-AS1*, *TRIM59*, *CDK6*, and *MSI2*. Detection of these structural alterations prompted diagnostic and/or risk reclassification in 5 cases.

**Conclusions:**

Optical genome mapping doubled the detection rate and improved the structural resolution of *MECOM*-r by identifying rearrangements incompletely resolved or missed by conventional cytogenetics. Implementation of OGM showed that *MECOM*-r can be cryptic and frequently overlooked, highlighting the value of high-resolution testing in HGMNs.

Key points

- Optical genome mapping (OGM) detected *MECOM* rearrangements in 12 of 252 (4.7%) high-grade myeloid neoplasms, doubling the number of detected cases by revealing 6 cases that were not detected by conventional diagnostic workflows based on karyotype and reflex fluorescence in situ hybridization (FISH).
- Detection of *MECOM* rearrangements exclusively by OGM led to reclassification of diagnosis and/or risk in 5 of 6 cases, often upgrading patients to a very poor risk category.
- Optical genome mapping revealed diverse and novel *MECOM* partner loci that were not resolvable by the standard karyotype and FISH workflow, including *GATA2*, *THADA*, *MYC*, *PHACTR1*, *MB21D2*, *XR_001744423.2*, *IL12A-AS1*, *TRIM59*, *CDK6*, and *MSI2*, providing new insights into the structural heterogeneity of *MECOM* rearrangements.

## INTRODUCTION

*MECOM* locus rearrangements define an infrequent but clinically important subset of adult acute myeloid leukemia (AML). These chromosomal alterations, most classically involving inv(3)(q21q26.2) or t(3;3)(q21;q26.2), relocate super-enhancer regions, including the *GATA2* distal hematopoietic enhancer (G2DHE), to the *MECOM* locus. These rearrangements result in *MECOM* overexpression, driving aggressive disease and poor outcomes.^1‐3^

Based on conventional karyotype and fluorescence in situ hybridization (FISH) analyses, *MECOM* rearrangements (*MECOM*-r) are expected to account for approximately 1% to 3% of adult AML cases globally, with a median age at diagnosis of 60 to 65 years.^4‐6^  *MECOM*-driven AML is high risk with a very poor prognosis and a reported 5-year survival rate of less than 15%.^2^^,^^7^ The International Consensus Classification and the fifth edition of the World Health Organization classifications consider AML with *MECOM*-r, irrespective of partner locus, a distinct disease-defining abnormality.^5^^,^^8^ Furthermore, according to European Leukemia Network 2022 criteria, *MECOM*-r is considered an adverse risk finding.^9^ Identification of *MECOM* involvement in cases otherwise considered intermediate risk has immediate therapeutic implications, supporting the use of intensified induction strategies and early consideration of allogeneic hematopoietic stem cell transplantation in first remission, which remains the only potentially curative approach for this high-risk disease.^1^^,^^7^^,^^9‐11^ Conventional karyotype and FISH analyses remain the first-line diagnostic tool for identifying *MECOM*-r. Karyotype analysis can identify large chromosomal alterations, including inv(3)(q21q26.2) and t(3;3)(q21;q26.2) events, while FISH can directly confirm *MECOM* locus rearrangements.^1^^,^^5^ However, these methods have important limitations. Karyotyping is constrained by a resolution of approximately 5 to 10 Mb and may miss subtle or complex rearrangements. Targeted FISH using a *MECOM* break-apart probe is sensitive and can confirm the presence of a *MECOM-*r, but upfront *MECOM* FISH testing is not routinely incorporated into the initial testing workflow in many laboratories, including our own, due to the high cost of this assay, which limits its use as a diagnostic screen in laboratories with funding or resource constraints. As a result, karyotypically cryptic *MECOM*-r can escape upfront detection using these conventional approaches, demanding the use of either broad-panel FISH or a more comprehensive, genome-wide approach.^5^^,^^12^^,^^13^

Optical genome mapping (OGM) is a transformative cytogenetic assay that detects structural and copy number variants ranging from approximately 500 bp up to large-scale chromosomal-level events.^14^ Recent multicenter studies in AML show OGM can reliably identify clinically relevant structural variants found by karyotype/FISH and reveal cryptic alterations.^14‐18^ Our experience with OGM as a first-line test in AML demonstrated strong performance in identifying diagnostically and prognostically relevant chromosome abnormalities, identifying approximately 18% more such variants over historical standard-of-care tests.^18^ Here, we present a sequential series of myeloid neoplasms collected during routine clinical analysis, retrospectively analyzed to characterize the spectrum of structural *MECOM*-r detectable by OGM and their impact on diagnosis and risk stratification.

## METHODS

All newly diagnosed AML and other high-grade myeloid neoplasms (HGMNs, defined as myeloid neoplasms with at least 10% myeloid blasts in blood or bone marrow) processed by our clinical laboratory between November 2023 and November 2025 were retrospectively analyzed. This cohort represents an unselected, consecutively collected series of cases referred for routine diagnostic evaluation. All samples morphologically compatible with a diagnosis of AML or other HGMNs were processed according to routine diagnostic workflows, including karyotype and OGM, with reflex FISH analysis performed at the discretion of laboratory geneticists. Clinical reports were retrospectively compiled for comparative evaluation, allowing a detailed assessment of structural variants affecting the *MECOM* locus and other co-occurring alterations detectable by karyotype, FISH, or OGM. Ethics approval for these research activities was granted by the University of British Columbia Clinical Research Ethics Board (H25-01143).

G-banded karyotype analysis was performed for all cases in accordance with routine, validated laboratory procedures. In short, bone marrow aspirate specimens collected in media or peripheral blood specimens collected in sodium heparin were processed upon receipt, including short-term culture and harvest, and then subjected to G-banded chromosome analysis. Findings were interpreted according to the International System for Human Cytogenomic Nomenclature (2020 or 2024) at the time of diagnosis.^19^^,^^20^

Optical genome mapping was performed in parallel for all cases on a separate, concurrently collected bone marrow aspirate or peripheral blood specimen in EDTA (preferred) or sodium heparin, according to validated, previously reported laboratory procedures.^18^ While all HGMN cases underwent conventional cytogenetic evaluation at diagnosis, OGM was performed for acute promyelocytic leukemias (APL) upon initial test launch but was discontinued at a later time point due to the absence of additional findings of clinical significance beyond the diagnostic abnormality identified by karyotype and FISH. Therefore, the cohort described includes a subset and not all APL cases diagnosed at our center during the inclusion time frame, with 10 confirmed diagnoses of APL lacking OGM, and thus excluded from the cohort of 262 specimens. As previously described by our laboratory,^18^ ultra-high molecular weight DNA was extracted using manufacturer protocols and run on the Bionano Saphyr or Stratys systems (Bionano Genomics), targeting a sample throughput of 1500 Gbp. Data quality and molecule metrics were assessed using Bionano Access software. Copy number variants and structural variants were identified using the Rare Variant Analysis pipeline until January 2025 (Access version 1.7.2 or 1.8.1, Solve version 3.7 or 3.8.1), when the improved Guided Assembly pipeline (Access version 1.8.2, Solve version 3.8.2) was verified and implemented clinically.^18^ Identified variants were filtered against a custom 273-gene pan-hematolymphoid panel^18^ and interpreted using a 4-tier, evidence-based categorization system adapted in our laboratory for OGM based on modified established consensus interpretation and reporting guidelines.^21^^,^^22^ Variant nomenclature was initially applied according to early published recommendations^23^ and transitioned to International System for Human Cytogenomic Nomenclature 2024 standards upon their release.^20^ Cryptic rearrangements were defined as structural variants detected by OGM that were absent or incompletely characterized on the corresponding G-banded karyotype.

A tricolor break-apart FISH probe set (CytoCell *MECOM* Tricolour Breakapart Probe; Oxford Gene Technology) was used as a reflex assay to confirm the presence of *MECOM*-r in cases showing evidence of chromosome 3 abnormalities by karyotype or as a reflex assay following *MECOM*-r identified by OGM to characterize the hybridization pattern by FISH. For 1 case, an additional break-apart FISH probe set (CytoCell *IKZF1* dual-color Breakapart Probe; Oxford Gene Technology) was used to confirm abnormalities on chromosome 7 associated with a *MECOM*-r. Fluorescence in situ hybridization assays were performed on the same cultured specimen used for karyotype analysis.

## RESULTS

**Figure 1:**
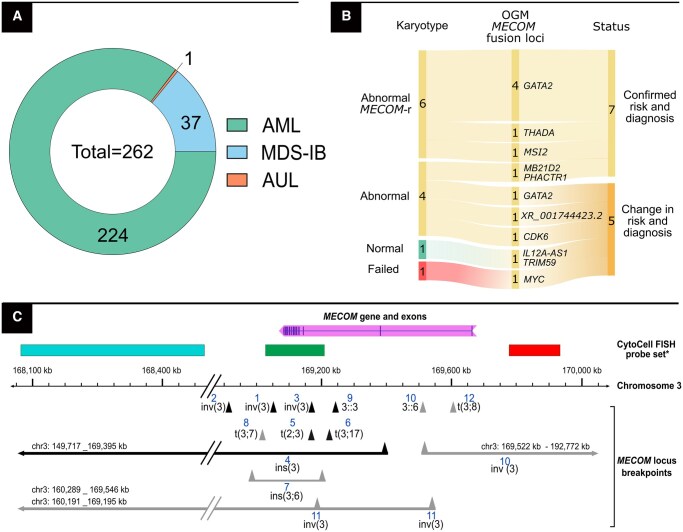
OGM-defined *MECOM*-r, diagnostic impact, and breakpoint mapping in high-grade myeloid neoplasms. (A) Distribution of 262 high-grade myeloid neoplasms in the study cohort, representing all consecutively collected cases of AML, AUL, and MDS-IB over a 2-year period. (B) Six *MECOM*-r cases identified by karyotype were confirmed and further characterized by OGM, which also detected 5 new cases and resolved 1 case with a failed karyotype, resulting in changes in diagnosis and risk stratification in 5 cases. (C) Schematic of the *MECOM* locus on chromosome 3, with the *MECOM* gene and exons indicated in fuchsia and purple, respectively. Optical genome mapping breakpoints for cases with chromosome 3 abnormalities identified by karyotype are indicated with black arrows. Optical genome mapping breakpoints in cases with *MECOM*-r that were missed by conventional karyotype analysis and detected by OGM are indicated by gray arrows. Case numbers are displayed in blue above the corresponding structural rearrangement event. AML, acute myeloid leukemia; AUL, acute undifferentiated leukemia; MDS-IB, myelodysplastic neoplasms with increased blasts; *MECOM*-r, *MECOM* rearrangements; OGM, optical genome mapping. *Approximate positions of CytoCell *MECOM* Breakapart FISH Probe set are shown based on publicly available manufacturer information.

The study cohort comprised 262 cases, with final diagnoses established after incorporating karyotype, FISH, and OGM result analyses. Most cases were defined as AML (87.5%, n = 224), followed by myelodysplastic neoplasms with increased blasts (MDS-IB) (14.1%, n = 37) and 1 acute undifferentiated leukemia (0.4%, n = 1) (Figure 1A). Of these, 252 cases were evaluated by OGM; 10 APL cases were not processed by OGM, as OGM was not performed for APL after an initial implementation period (see Methods section). *MECOM*-r were identified in 12 cases (4.7%; median age 63 years), with equal representation of male and female patients (n = 6 each) (Figure 1B, C; Table S1).

### Cases 1-6: Cases with karyotypically identifiable rearrangements involving *MECOM*

**Figure 2:**
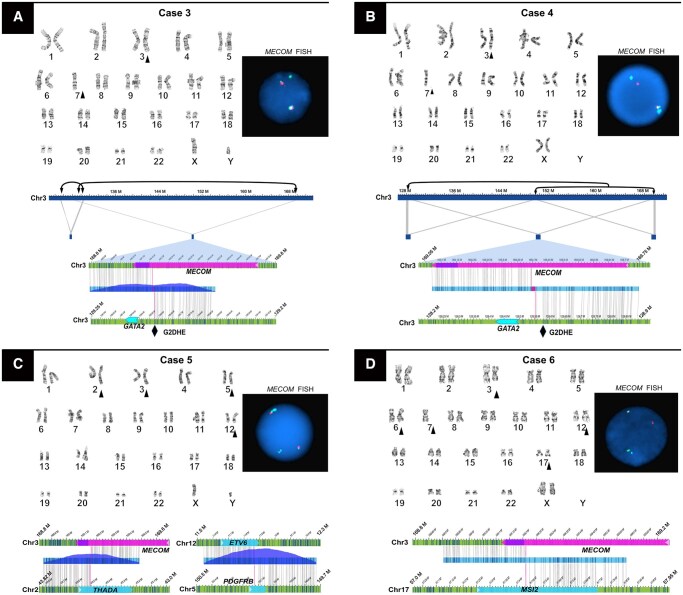
*MECOM*-r identified by karyotype analysis and characterized by OGM. (A) Case 3. G-banded karyotype shows an inversion of chromosome 3 between 3q21 and 3q26.2. Interphase FISH (inset) confirms *MECOM* involvement, separating aqua-green and green-red signals. Optical genome mapping reveals overlapping inversions on chromosome 3 from 3q21.2 to 3q22.1 and from 3q21.3 to 3q26.2; the approximate breakpoints of these inversions are indicated with connected black arrows. Detailed variant maps show the resulting *GATA2*::*MECOM* fusion and relocation of the G2DHE downstream of *MECOM*. (B) Case 4. G-banded karyotype shows an inversion of chromosome 3 between 3q21 and 3q26. Interphase FISH (inset) confirms *MECOM* involvement, showing separation of the aqua-green signals from the red signal. Optical genome mapping reveals a large intrachromosomal insertion of chromosome 3 material 3q25.1-3q26.2 into 3q21.3, resulting in a *GATA2*::*MECOM* fusion and relocation of G2DHE downstream of *MECOM*. (C) Case 5. G-banded karyotype shows a translocation with breakpoints at 2p21 and 3q26 and a second translocation with breakpoints at 5q33 and 12p13. Interphase FISH (inset) confirms *MECOM* involvement, separating aqua-green and green-red signals. Detailed variant maps resolve a *THADA*::*MECOM* fusion from t(2;3) and a *PDGFRB*::*ETV6* fusion from t(5;12). (D) Case 6. G-banded karyotype shows additional material on chromosomes 3q26 and 17q25. Interphase FISH (inset) confirms *MECOM* involvement, showing separation of the aqua-green signals from the red signal. Optical genome mapping reveals an *MSI2*::*MECOM* fusion resulting from the t(3;17) rearrangement. Abnormalities identified in karyotype analysis are indicated with black triangles and detailed in Table S1. FISH, fluorescence in situ hybridization; G2DHE, *GATA2* distal hematopoietic enhancer; *MECOM*-r, *MECOM* rearrangements; OGM, optical genome mapping.

Four individuals had inversion of the long arm of chromosome 3 with breakpoints at 3q21 and 3q26.2 by karyotype. Fluorescence in situ hybridization confirmed rearrangement of the *MECOM* locus, and OGM showed that these inversions resulted in *GATA2::MECOM* fusions, resulting from simple 2-break (cases 1 and 2) or more complex (cases 3 and 4) rearrangements. Cases 1, 3, and 4 also showed monosomy 7, among other findings. Integration of OGM in these 4 cases maintained the classification and stratification as AML with *MECOM*-r and high-risk status. In case 1, karyotype identified a simple inversion on chromosome 3 from 3q21 to 3q26.2, with OGM confirming a *GATA2::MECOM* fusion (Figure S1A). Case 2 was initially associated with a diagnosis of MDS with an isoderivative chromosome 20q identified by karyotype. Additional cytogenetic abnormalities were identified upon progression by karyotype, FISH, and OGM, including a simple inversion of chromosome 3 at 3q21.3 and 3q26.2, resulting in a *GATA2::MECOM* fusion and an interstitial deletion of 5q with breakpoints at 5q22 and 5q33 (Figure S1B). In case 3, karyotype showed an inversion of chromosome 3 with breakpoints at 3q21 and 3q26.2. Optical genome mapping confirmed the inversion and revealed an additional smaller nested inversion with breakpoints at 3q21.2 and 3q22.1, resulting in a *GATA2::MECOM* fusion (Figure 2A). In case 4, karyotype showed an inversion on the long arm of chromosome 3 from 3q21 to 3q26. Optical genome mapping revealed a complex intrachromosomal rearrangement involving 3 breakpoints, with a large insertion of material from 3q25.1-3q26.2 into 3q21.3, resulting in a *GATA2::MECOM* fusion (Figure 2B).

Two other individuals had karyotypes showing rearrangements involving the *MECOM* locus. Integration of OGM in these 2 analyses maintained the classification and stratification as AML with *MECOM*-r with high risk. In case 5, the karyotype demonstrated translocations between chromosomes 2 and 3 with breakpoints at 2p21 and 3q26, as well as between chromosomes 5 and 12 at 5q33 and 12p13. Fluorescence in situ hybridization confirmed involvement of the *MECOM* locus, and OGM further resolved the rearrangements as *THADA*::*MECOM* and *PDGFRB*::*ETV6* fusions, respectively (Figure 2C). Optical genome mapping identified an additional inversion on chromosome 3 from q26.31 to 3q29, with deletions of material at 3q26.31-3q26.32 and 3q28-3q29 (Table S1). Case 6 had a complex karyotype with monosomy 7, including additional material on chromosome 3 at 3q26 and additional material on chromosome 17 at 17q25. Fluorescence in situ hybridization confirmed the presence of a *MECOM*-r, and OGM identified a rearrangement between chromosomes 3 and 17 at 3q26.2 and 17q22, resulting in an *MSI2::MECOM* fusion (Figure 2D).

### Cases 7-12: Cases with *MECOM*-r missed by conventional karyotype analysis and detected by OGM

**Figure 3:**
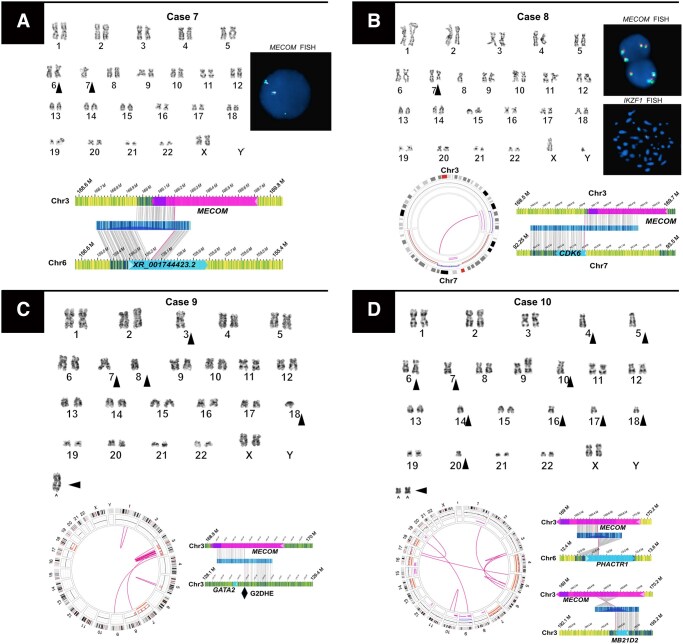
*MECOM*-r missed by conventional karyotype and detected by OGM. (A) Case 7. G-banded karyotype shows monosomy 7 and additional material on chromosome 6p23. Interphase reflex FISH (top inset) confirms *MECOM* involvement with a green signal separated from aqua-green-red signals. Detailed variant maps show an insertion of *MECOM* into chromosome 6, resulting in an *XR_001744423.2*::*MECOM* loci fusion. (B) Case 8. G-banded karyotype shows a derivative chromosome 7 with additional material at 7p12 and 7q22. Interphase reflex *MECOM* FISH (top inset) shows 1 cell with 3 copies (bottom cell) of the *MECOM* locus and another with 4 copies (top cell). Metaphase FISH for *IKZF1* (bottom inset) confirms chromosome 7 involvement. Circos plot and detailed variant maps show a rearrangement between chromosomes 3 and 7, resulting in a *CDK6*::*MECOM* fusion, with gain of 3q26.2-3q29 and losses of 7p distal to p12 and 7q distal to q21.2. (C) Case 9. G-banded karyotype shows a complex karyotype with multiple monosomies and marker chromosomes of unknown origin. Circos plot and detailed variant maps show apparent chromothripsis of chromosome 3, resulting in a *GATA2*::*MECOM* fusion. Fluorescence in situ hybridization was not performed. (D) Case 10. G-banded karyotype shows a hypodiploid complex karyotype with multiple monosomies. Optical genome mapping Circos plot shows additional structural complexity. Detailed variant maps show 2 *MECOM* rearrangements on chromosome 3, resulting in *MB21D2*::*MECOM* and *PHACTR1*::*MECOM* loci fusions. Fluorescence in situ hybridization was not performed. Abnormalities identified in karyotype analysis are indicated with black triangles and detailed in Table S1. FISH, fluorescence in situ hybridization; *MECOM*-r, *MECOM* rearrangements; OGM, optical genome mapping.

For 5 individuals (cases 7-11), karyotype analysis showed multiple structural abnormalities but did not suggest rearrangement of chromosome 3, but OGM identified *MECOM*-r in all cases, leading to diagnostic reclassification and risk reclassification in most cases. In case 7, karyotype showed monosomy 7 and additional material on chromosome 6 at 6p23. Optical genome mapping resolved the structure of chromosome 6p, showing an inversion between 6p22.3 and 6p21.2 with additional material from 6p21.32 to 6p12.3. Optical genome mapping also identified a focal 0.24 Mb insertion of chromosome 3q26.2 (including *MECOM*) into chromosome 6q25.3, resulting in an *XR_001744423.2::MECOM* loci fusion. This rearrangement supported the reclassification from MDS-IB2 to AML with *MECOM*-r and increased risk (Figure 3A). In case 8, karyotype demonstrated a derivative chromosome 7 with additional material at 7p12 and 7q22, resulting in terminal deletions distal to both breakpoints, in the context of a clinical history consistent with a therapy-related myeloid neoplasm. Fluorescence in situ hybridization analysis identified a deletion of 7p, including the *IKZF1* locus, but did not resolve the full extent of the rearrangement. Optical genome mapping clarified the underlying structural complexity by identifying a rearrangement between chromosome 3q26.2 and 7q21.2, resulting in a *CDK6*::*MECOM* fusion, accompanied by gain of 3q26.2 to 3q29 and loss of 7q distal to the breakpoint at q21.2. Reflex *MECOM* FISH confirmed a single rearranged allele in approximately 40% of cells and an additional copy of the rearranged allele in approximately 36% of cells. Together, these findings supported reclassification to therapy-related AML with *MECOM*-r and increased risk (Figure 3B). Case 9 presented a complex karyotype with monosomies of chromosomes 3, 7, 8, and 18 and marker chromosomes of unknown origin. Optical genome mapping identified complex patterns consistent with chromothripsis of chromosome 3, resulting in a *GATA2::MECOM* fusion involving 3q21.3 and 3q26.2. Optical genome mapping identified additional interstitial deletions from 5q31.1 to 5q32 on chromosome 5 and between 8p23.2 and 8p21.2 on chromosome 8 (Figure 3C). These findings supported the reclassification from myelodysplasia-related acute myeloid leukaemia (AML-MR) to AML with *MECOM*-r without a change in overall risk. In case 10, the karyotype showed a complex monosomal karyotype, including loss of chromosome 7. Optical genome mapping showed 2 putative *MECOM*-r on chromosome 3, including an inversion from 3q26.2 to 3q29, resulting in a fusion between the *MB21D2* locus and *MECOM*. A second event involved a translocation between the rearranged 3q26.2 breakpoint and chromosome 6 at 6p24.1, resulting in a fusion involving the *PHACTR1* locus and *MECOM*. Optical genome mapping revealed additional rearrangements across multiple chromosomes, along with a focal loss at 20q22.12 (Figure 3D). This high-risk genomic profile is compatible with the classification of a blast phase myeloproliferative neoplasm (MPN) harboring abnormalities associated with a very poor prognosis in myeloid malignancies.

**Figure 4:**
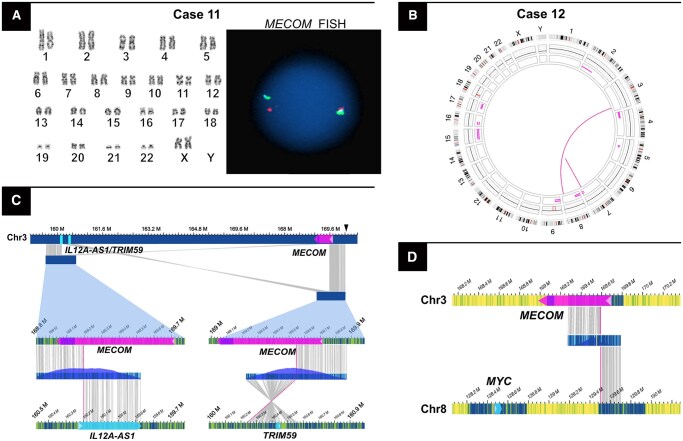
*MECOM*-r detected by OGM in cases with normal or failed karyotypes. (A) Case 11. G-banded karyotype was normal. Interphase reflex *MECOM* FISH (inset) shows *MECOM* locus involvement. Detailed variant maps reveal 2 cryptic inversions on chromosome 3, resulting in an *IL12A*-*AS1*/*TRIM59*::*MECOM* loci fusion. (B) Case 12. G-banded karyotype failed due to insufficient metaphases. Optical genome mapping shows a rearrangement between chromosomes 3 and 8 at 3q26.2 and 8q24.21, resulting in a *MYC*::*MECOM* fusion. FISH, fluorescence in situ hybridization; *MECOM*-r, *MECOM* rearrangements; OGM, optical genome mapping.

In the remaining 2 cases, karyotypes were normal or failed, yet OGM identified *MECOM-*r in both cases, leading to diagnostic reassessment and risk reclassification. In case 11, karyotype showed no abnormalities, but OGM revealed cryptic inversions on chromosome 3 from 3q25.33 to 3q26.2, confirmed by reflex FISH, resulting in a fusion between the *IL12A-AS1/TRIM59* loci and *MECOM* (Figure 4A). An additional rearrangement between chromosomes 12 and 15 at 12p13.2 and 15q25.3 resulted in a *ETV6::NTRK3* fusion (Table S1). Altogether, these findings supported the reclassification of this case from AML-MR to AML with *MECOM-*r with an associated increased risk. In case 12, insufficient metaphases were obtained for karyotype analysis, and a failed result was issued. Optical genome mapping was therefore critical for this case assessment, as it not only yielded a result but also identified a rearrangement between chromosomes 3 and 8 at 3q26.2 and 8q24.21, resulting in a *MYC::MECOM* fusion, enabling the classification of this case as AML with *MECOM*-r and supporting an increased risk level (Figure 4B).

## DISCUSSION

In this unselected cohort of AML and other HGMNs, defined as myeloid neoplasms with a blast count of 10% or more in bone marrow or peripheral blood, *MECOM*-r were identified in 4.7% of cases (12/252). This prevalence exceeded the 1% to 3% typically reported in studies that rely on karyotype to detect abnormalities involving this locus,^6^^,^^7^ suggesting that the true incidence of *MECOM*-r has been historically underestimated due to the limited resolution of conventional cytogenetic approaches. In our cohort, only half of *MECOM*-r cases (6/12) were detected by standard methods, underscoring the substantial incremental diagnostic yield provided by genome-wide, high-resolution OGM. While *MECOM* FISH was not included in our upfront diagnostic workflow, this test could have facilitated detection of some cryptic *MECOM*-r cases, particularly in the setting of monosomy 7, a recurrent cytogenetic abnormality known to be associated with *MECOM*-r in a subset of cases, as seen in cases 6, 7, and 9.

The central pathogenic mechanism underlying neoplasms with *MECOM*-r is proposed to be enhancer hijacking, whereby distal regulatory elements are repositioned to aberrantly activate the *MECOM* (EVI1) promoter.^2^^,^^24^ In our cohort, rearrangements involving the *GATA2/RPN1* locus were most frequent (5/12), including a cryptic *MECOM*-r. These events are known to result in a functional double-hit mechanism, combining ectopic activation of *MECOM* through relocation of G2DHE with concomitant disruption and haploinsufficiency of the *GATA2* gene itself.^1‐3^ Optical genome mapping provided enhanced structural resolution by delineating the orientation and spatial configuration of these rearrangements. While conventional cytogenetics captured large-scale positional changes, when evident, OGM enabled precise mapping of enhancer repositioning relative to the *MECOM* promoter locus. These configurations are consistent with the disruption of insulating topologically associating domain boundaries, physically approximating the G2DHE element to the *MECOM* promoter.^2^^,^^25^

Importantly, OGM resolved structurally diverse rearrangements involving atypical partner loci, including *MSI2*, *CDK6*, *MYC*, and *THADA*, which harbor potent regulatory elements capable of functionally recapitulating the transcriptional consequences of inv(3)/t(3;3) and have been reported to be associated with *MECOM* overexpression.^25‐27^ These findings support a unifying model in which diverse structural variants converge on a shared biological mechanism of *MECOM* activation through enhancer hijacking, despite marked heterogeneity in their genomic architecture.^2^^,^^24^ Several putative partner loci identified in this cohort, including *MB21D2*, *PHACTR1*, *XR_001744423.2*, and *IL12A-AS1*/*TRIM59*, remain poorly characterized in hematologic malignancies. Their detection further highlights the ability of OGM to resolve *MECOM*-r involving atypical or noncanonical genomic regions.

Beyond canonical inv(3)(q21q26.2) and t(3;3)(q21;q26.2), *MECOM*-r arose through a variety of structurally complex rearrangements that were often missed or incompletely characterized by conventional methods. These included multi-breakpoint intrachromosomal or interchromosomal rearrangements, nested inversions with focal deletions, chromothripsis of chromosome 3, and intragenic *MECOM* insertions. Importantly, many of these abnormalities would likely have been interpreted as canonical, unresolved by karyotype alone, or entirely missed, highlighting the added value of OGM in resolving breakpoint architecture at the *MECOM* locus. Furthermore, the culture-independent nature of OGM provides a key technical advantage, enabling genomic characterization even in the absence of viable metaphases, as illustrated by case 12, and consistent with our previous experience in cases where OGM yielded a result while karyotype failed or performed suboptimally.^18^

The clinical implications of identifying these cryptic and atypical *MECOM*-r are substantial, as OGM findings improved detection of *MECOM*-r, enabled refined genomic characterization, and supported more accurate risk stratification. The prevalence observed in this cohort further suggests that *MECOM*-r may be underrecognized when assessed using conventional cytogenetic approaches. Collectively, these findings indicate that accurate identification of *MECOM*-r requires high-resolution, genome-wide interrogation, as clinically significant rearrangements may remain undetected or incompletely characterized using standard methods.

## Supplementary Material

aqag087_Supplementary_Data

## Data Availability

The results analyzed during this study are available in this manuscript and its supplementary material. Additional data are available from the corresponding author upon reasonable request.

## References

[1] Grimwade D , Ivey A, Huntly BJP. Molecular landscape of acute myeloid leukemia in younger adults and its clinical relevance. Blood. 2016;127:29-41. doi:10.1182/blood-2015-07-604496 26660431 PMC4705608

[2] Gröschel S , Sanders MA, Hoogenboezem R, et al A single oncogenic enhancer rearrangement causes concomitant EVI1 and GATA2 deregulation in leukemia. Cell. 2014;157:369-381. doi:10.1016/j.cell.2014.02.019 24703711

[3] Yamazaki H , Suzuki M, Otsuki A, et al A remote GATA2 hematopoietic enhancer drives leukemogenesis in inv(3)(q21; q26) by activating EVI1 expression. Cancer Cell. 2014;25:415-427. doi:10.1016/j.ccr.2014.02.008 24703906 PMC4012341

[4] De Braekeleer M , Le Bris MJ, De Braekeleer E, Basinko A, Morel F, Douet-Guilbert N. 3q26/ EVI1 rearrangements in myeloid hemopathies: a cytogenetic review. Future Oncol. 2015;11:1675-1686. doi:10.2217/fon.15.64 26043219

[5] Khoury JD , Solary E, Abla O, et al The 5th edition of the World Health Organization classification of haematolymphoid tumours: myeloid and histiocytic/dendritic neoplasms. Leukemia. 2022;36:1703-1719. doi:10.1038/s41375-022-01613-1 35732831 PMC9252913

[6] Lazarevic V , Hörstedt AS, Johansson B, et al Incidence and prognostic significance of karyotypic subgroups in older patients with acute myeloid leukemia: the Swedish population-based experience. Blood Cancer J. 2014;4:e188. doi:10.1038/bcj.2014.10 24583534 PMC3944658

[7] Lugthart S , Gröschel S, Beverloo HB, et al Clinical, molecular, and prognostic significance of WHO Type inv(3)(q21q26.2)/t(3; 3)(q21; q26.2) and various other 3q abnormalities in acute myeloid leukemia. J Clin Oncol. 2010;28:3890-3898. doi:10.1200/JCO.2010.29.2771 20660833

[8] Arber DA , Orazi A, Hasserjian RP, et al International Consensus Classification of myeloid neoplasms and acute leukemias: integrating morphologic, clinical, and genomic data. Blood. 2022;140:1200-1228. doi:10.1182/blood.2022015850 35767897 PMC9479031

[9] Döhner H , Wei AH, Appelbaum FR, et al Diagnosis and management of AML in adults: 2022 recommendations from an international expert panel on behalf of the ELN. Blood. 2022;140:1345-1377. doi:10.1182/blood.2022016867 35797463

[10] Joshi U , Shallis RM. MECOM-rearranged acute myeloid leukemia: pathobiology and management strategies. Hematol Rep. 2025;17:59. doi:10.3390/hematolrep17060059 41283235 PMC12642003

[11] Richard-Carpentier G , Rausch CR, Sasaki K, et al Characteristics and clinical outcomes of patients with acute myeloid leukemia with inv(3)(q21q26.2) or t(3; 3)(q21; q26.2). Haematologica. 2023;108:2331-2342. doi:10.3324/haematol.2022.282030 36951163 PMC10483357

[12] Baldazzi C , Luatti S, Zuffa E, et al Complex chromosomal rearrangements leading to MECOM overexpression are recurrent in myeloid malignancies with various 3q abnormalities. Genes Chromosomes Cancer. 2016;55:375-388. doi:10.1002/gcc.22341 26815134

[13] Tang Z , Wang W, Yang S, et al 3q26.2/MECOM rearrangements by pericentric inv(3): diagnostic challenges and clinicopathologic features. Cancers (Basel). 2023;15:458. doi:10.3390/cancers15020458 36672407 PMC9856433

[14] Smith AC , Neveling K, Kanagal‐Shamanna R. Optical genome mapping for structural variation analysis in hematologic malignancies. Am J Hematol. 2022;97:975-982. doi:10.1002/ajh.26587 35560245

[15] Dremsek P , Schwarz T, Weil B, Malashka A, Laccone F, Neesen J. Optical genome mapping in routine human genetic diagnostics—its advantages and limitations. Genes (Basel). 2021;12:1958. doi:10.3390/genes12121958 34946907 PMC8701374

[16] Levy B , Baughn LB, Akkari Y, et al Optical genome mapping in acute myeloid leukemia: a multicenter evaluation. Blood Adv. 2023;7:1297-1307. doi:10.1182/bloodadvances.2022007583 36417763 PMC10119592

[17] Levy B , Kanagal-Shamanna R, Sahajpal NS, et al A framework for the clinical implementation of optical genome mapping in hematologic malignancies. Am J Hematol. 2024;99:642-661. doi:10.1002/ajh.27175 38164980

[18] Parlow JNC , Hamadeh Z, Salcedo-Porras N, AlBulushi F, McGinnis E, Spence T. Technical validation and prospective clinical utility of optical genome mapping in acute leukemia workup: insights from 200 cases following deployment as a first-line diagnostic test in a tertiary care center. J Mol Diagn. 2026;28:377-388. doi:10.1016/j.jmoldx.2026.01.002 41571091

[19] ISCN. ISCN 2020: An International System for Human Cytogenomic Nomenclature (2020). Reprint of “Cytogenetic and Genome Research 2020, Vol. 160, No. 7-8.” 1st ed. S. Karger AG; 2020.

[20] ISCN. ISCN 2024—an international system for human cytogenomic nomenclature (2024). Cytogenet Genome Res. 2024;164:1-224. doi:10.1159/000538512 39571546

[21] Li MM , Datto M, Duncavage EJ, et al Standards and guidelines for the interpretation and reporting of sequence variants in cancer. J Mol Diagn. 2017;19:4-23. doi:10.1016/j.jmoldx.2016.10.002 27993330 PMC5707196

[22] Mikhail FM , Biegel JA, Cooley LD, et al Technical laboratory standards for interpretation and reporting of acquired copy-number abnormalities and copy-neutral loss of heterozygosity in neoplastic disorders: a joint consensus recommendation from the American College of Medical Genetics and Genomics (ACMG) and the Cancer Genomics Consortium (CGC). Genet Med. 2019;21:1903-1916. doi:10.1038/s41436-019-0545-7 31138931

[23] Moore S , McGowan-Jordan J, Smith AC, et al; ISCN Standing Committee. Genome mapping nomenclature. Cytogenet Genome Res. 2023;163:236-246. doi:10.1159/000535684 38071973

[24] Ottema S , Mulet-Lazaro R, Erpelinck-Verschueren C, et al The leukemic oncogene EVI1 hijacks a MYC super-enhancer by CTCF-facilitated loops. Nat Commun. 2021;12:5679. doi:10.1038/s41467-021-25862-3 34584081 PMC8479123

[25] Ottema S , Mulet-Lazaro R, Beverloo HB, et al Atypical 3q26/MECOM rearrangements genocopy inv(3)/t(3; 3) in acute myeloid leukemia. Blood. 2020;136:224-234. doi:10.1182/blood.2019003701 32219447

[26] De Weer A , Speleman F, Cauwelier B, et al; Groupe Français de Cytogénétique Hématologique (GFCH). EVI1 overexpression in t(3; 17) positive myeloid malignancies results from juxtaposition of EVI1 to the MSI2 locus at 17q22. Haematologica. 2008;93:1903-1907. doi:10.3324/haematol.13192 18815193

[27] Tang G , Hu S, Wang SA, et al t(3; 8)(q26.2; q24) often leads to MECOM/MYC rearrangement and is commonly associated with therapy-related myeloid neoplasms and/or disease progression. J Mol Diagn. 2019;21:343-351. doi:10.1016/j.jmoldx.2018.10.005 30576868

