# Supplementary material for: Optical genome mapping refines diagnosis of high-grade myeloid neoplasms through detection of MECOM rearrangements cryptic to conventional karyotype
Source: Am J Clin Pathol. 2026 Aug 4;166(2):aqag087. doi: 10.1093/ajcp/aqag087 (PMC13437022; doi:10.1093/ajcp/aqag087)
Supplement: Supplementary file 1 [file aqag087_supplementary_data.docx]

| **Supplementary Table 1.** **Summary of karyotype, FISH, and OGM findings and their impact on classification and risk.** Cases are described by sex and age, with G-banded karyotype, interphase *MECOM* FISH results, and OGM findings reported in ISCN format. OGM-informed classification indicates cases where OGM findings refined or changed the diagnostic classification. OGM-informed risk indicates cases where OGM findings impacted risk stratification. Abbreviations: fluorescence in situ hybridization (FISH); optical genome mapping (OGM); International System for Human Cytogenomic Nomenclature (ISCN). | | | | | | |
| --- | --- | --- | --- | --- | --- | --- |
| **Case ID** | **Sex and Age** | **Karyptype** | **FISH** | **OGM ISCN** | **OGM Informed Classification?** | **OGM Informed risk?** |
| **1** | M 71 | 45,XY,inv(3)(q21q26.2),-7[10] | nuc ish(D3S1614,EVI1,D3S3523)x2(D3S1614 sep EVI1/D3S3523x1)[63/100] | ogm[GRCh38] (X,Y)x1,inv(3)(q21.3q26.2)(128,582,573-128,585,738_169,052,490)[0.88],(7)x1[0.86] | NO | NO |
| 2 | M 74 | 46,XY,ider(20)(q10)del(20)(q11.2q13.2)[4]/46,idem,inv(3)(q21q26.2),del(5)(q22q33)[21] | nuc ish(D3S1614,EVI1,D3S3523)x2(D3S1614 sep EVI1/D3S3523x1)[49/100] | ogm[GRCh38] (X,Y)x1,inv(3)(q21.3q26.2)(128,552,431-128,557,325_168,908,829-168,913,964)(GATA2::MECOM)[0.61],(5)(q14.3q35.3)(86,870,458_179,298,916)x1-2[0.58],(9)(q21.11q34.13)(68,298,843_132,996,963)x2-3[0.62],(20)(p13p11.1)(70,156_25,742,741)x1-2[0.88],(20)(q11.21)(31,182,877_32,497,427)x3-4[>0.99],(20)(q11.21q13.31)(32,512,803_57,334,858)x1-2[0.97],(20)(q13.31q13.33)(57,343,224_61,861,320)x3-4[>0.99] | NO | NO |
| **3** | M 60 | 45,XY,inv(3)(q21q26.2),-7[9]/46,XY[1] | nuc ish(D3S1614,EVI1,D3S3523)x2(D3S1614 con EVI1 sep EVI1 con D3S3523x1)[47/100] | ogm[GRCh38] (X,Y)x1,der(3)inv(3)(q21.2q22.1)(125,930,850_130,096,950)inv(3)(q21.3q26.2)(128,557,325_169,173,110)(GATA2::MECOM)[0.80],-7[0.81] | NO | NO |
| **4** | F 38 | 45,XX,?inv(3)(q21q26),-7[9]/46,XX[1] | nuc ish(D3S3523,EVI1,D3S1614)x2(D3S3523 sep EVI1/DS1614x1)[67/100] | ogm[GRCh38] (X)x2,ins(3)(pter_q21.3::q25.1_q26.2::q21.3_q25.1::q26.2_qter)(pter_128,542,236::149,717,760_169,395,983::128,552,431_149,699,603::169,401,245_qter)(RPN1::MECOM)[0.81],(7)x1[0.76] | NO | NO |
| **5** | M 63 | 46,XY,t(2;3)(p21;q26),t(5;12)(q33;p13) | nuc ish(D3S1614,EVI1,D3S3523)x2(D3S1614/EVI1 sep EVI1/D3S3523x1)[141/200] | ogm[GRCh38] (X,Y)x1,t(2;3)(p21;q26.2)(43,499,788-4,3506,033;169,165,150.5)(MECOM::THADA)[0.80],der(3)inv(3)(q26.31q29)(175,638,547_192,805,140)del(3)(q26.31q26.32)(175,638,547_179,034,652)del(3)(q28q29)(189,101,565_192,805,140)[0.33],t(5;12)(q32;p13.2)(150,126,003_150,154,881;11,865,465_11,881,907)(PDGFRB::ETV6)[0.69] | NO | NO |
| **6** | F 57 | 45,XX,add(3)(q26),-7,add(17)(q25)[4]/45,idem,add(6)(q13),?add(12)(q24)[16] | nuc ish(D3S3523,EVI1,D3S1614)x2(D3S3523 sep EVI1/D3S1614)[96/100] | ogm[GRCh38] (X)x2,t(3;17)(q26.2;q22)(169,211,131;57,433,782)(MECOM::?MSI2)[0.74],6q12q23.3(65,933,392_138,072,506)x1[0.76],(7)x1[0.97] | NO | NO |
| **7** | F 59 | 45,XX,add(6)(p23),-7[10] | nuc ish(D3S3364/MECOM/D3S3523x2,MECOMx1)[41/100] | ogm[GRCh38] (X)x2,inv(6)(p22.3;p21.2)(23446266-23455996.5_36678067-36976632)[0.30],dup(6)(p21.32p12.3)(29,425,299_47,231,526)[0.32],ins(6;3)(q25.3;q26.2q26.2)(156,164,757-156,171,976;168,964,269_169,200,949)(XR_001744423.2::MECOM)[0.24],-7[0.29] | YES | YES |
| **8** | M 80 | 46,XY,der(7)add(7)(p12)add(7)(q22)[11] | nuc ish(IKZF1x1,D7S2014x2)[46/100]/ish(D3S1614x2,EVI1x4,D3S3523x4)(EVI1 con D3S3523x2)[40/100]/(D3S1614x2,EVI1x3,D3S3523x3)(EVI1 con D3S3523x1)[36/100] | ogm[GRCh38] (X,Y,)x1,t(3;7)(q26.2;q21.2)(?169,043,134;?92,786,069)(CDK6::MECOM)[?],3q26.2q29(169,043,134_198,230,596)x4[0.62],7p22.3p11.2(1,633,048_58,027,799)x1[0.49],7q11.21q21.2(62,910,762_92,781,839)x3[0.53],7q21.2q36.3(92,786,069_159,334,984)x1[0.74]. | YES | YES |
| **9** | F 63 | 43,XX,-3,-7,-8,-18,+mar[10] | Not Performed | ogm[GRCh38] (X)x2,(3)cth,3::3(q21.3::q26.2)(128,585,738::169,242,996)(MECOM::RPN1)[0.82],5q31.1q32(131,542,774_147,445,358)x1[0.84],(7)x1[0.91],8p23.2p21.2(3,379,021_23,850,243)x1[0.84],(18)x1[0.91] | YES | NO |
| **10** | F 75 | 39,XX,-4,-5,add(6)(p22),-7,add(10)(q21),-14,-16,-17,-18,-20,+2mar[cp3]/46,XX[2] | Not Performed | ogm[GRCh38] (X)x2,der(1)t(1;5)(q42.12;q33.1)(226,001,036;151,775,609)[0.38],inv(3)(q26.2;q29)(169,522,413;192,772,004)[0.76],3::6(q26.2;p24.1)(169,514,229;12,886,824)(MECOM::PHACTR1)[0.78],(4)x1[0.79],(5)x1[0.68],(7)x1[0.74],t(10;10)(q21.1;q25.1)(53,709,799;106,439,745)[0.74],(14)x1[0.75],(16)x1[0.69],(17)x1[0.69],(18)x1[0.79],der(19)t(9;19)(p13.3;p13.3)(34,011,515;3,369,841)[0.38],der(20)t(17;20)(q22;q11.23)(58,453,541;37,057,112)[0.60],21q22.12(34,568,048_35,281,400)x1(RUNX1-)[0.79] | NO | YES |
| **11** | F 59 | 46,XX[20] | nuc ish(D3S1614,EVI1,D3S3523)x2(D3S1614/EVI1 sep D3S3523)[51/200] | ogm[GRCh38] (X)x2,inv(3)(q25.33q26.2)(160,191,585-160,289,824_169,195,566-169,546,255)(IL12A-AS1 or TRIM59-IFT80::MECOM)[0.66],t(12;15)(p13.2;q25.3)(11,868,950;87,992,074)(ETV6::NTRK3)[0.58] | YES | YES |
| **12** | M 91 | Failed | Not Performed | ogm[GRCh38] (X,Y)x1,t(3;8)(q26.2;q24.21)(169,587,924_169,625,417;129,504,351_129,739,068)(MECOM::MYC)[0.58] | YES | YES |
| *ISCN nomenclature was copied directly from the original clinical reports, which followed early OGM published recommendations ^23^, with nomenclature transitioning to the 2020^19^ and 2024^20^ ISCN guidelines at the time of reporting. | | | | | | |

**Supplementary Figure 1. Canonical inv(3)(q21q26.2) cases demonstrating karyotype, FISH, and OGM findings.** G-banded karyotypes showed an inversion of chromosome 3 from 3q21.3 to 3q26.2, representing a canonical inv(3) in (A) Case 1 and (B) Case 2. Interphase *MECOM* FISH (insets) showed separation of the aqua signal from the green-red signals in both cases, confirming *MECOM* involvement. OGM variant maps showed a segment of chromosome 3 with a detailed view of the inversion, including relocation of the G2DHE (black diamond) downstream of *MECOM*. Abnormalities identified in karyotype analysis are indicated with black triangles and detailed in Supplementary Table 1. Abbreviations: Fluorescence in situ Hybridization (FISH); *GATA2* distal hematopoietic enhancer (G2DHE); Optical Genome Mapping (OGM).
